# The effects of the coronavirus disease pandemic on intravenous thrombolytic therapy among patients with acute ischemic stroke in Dalian, China

**DOI:** 10.1186/s12883-023-03053-5

**Published:** 2023-01-09

**Authors:** Xin Pan, Shubei Ma, Xiaowen Sui, Lili Xie, Furong Li, Zhengping Cheng, Li Cui, Hongling Zhao

**Affiliations:** 1 Department of Neurology, Dalian Municipal Central Hospital Affiliated With Dalian Medical University, No. 826, Southwest Road, Shahekou District, Dalian, 116033 Liaoning Province China; 2 Stroke Center, Dalian Municipal Central Hospital Affiliated With Dalian Medical University, Dalian, 116033 Liaoning Province China

**Keywords:** Acute ischemic stroke, COVID-19, Intravenous thrombolytic therapy, Stroke emergency maps, Green channel

## Abstract

**Background:**

We investigated the influence of the coronavirus disease 2019 (COVID-19) pandemic on the number of patients with acute ischemic stroke who received intravenous thrombolytic therapy (ITT) in Dalian, China, in 2020.

**Methods:**

This retrospective descriptive study, conducted from February 1, 2020, to August 31, 2020, examined 13 hospitals in Dalian that participated in the “stroke emergency map”. To use this “stroke emergency map” of China, patients followed the official “Stroke Map” WeChat account and dialed 120 for emergency medical services. We analyzed the number of patients with acute ischemic stroke who underwent ITT. In particular, we examined the onset-to-door time (ODT), door-to-needle time (DNT), onset-to-needle time (ONT), mode of transportation to the hospital, and National Institutes of Health Stroke Scale (NIHSS) scores before and after ITT. Data were collected for the aforementioned period and compared with the 2021 baseline data from the same time of year. The Mann‒Whitney U test was performed for data analysis.

**Results:**

Compared with the data from 2020, the number of patients with acute ischemic stroke who underwent ITT increased (from 735 to 1719 cases) in 2021, but the DNT decreased (from 59 to 45 min; *P* = 0.002). Moreover, 83.9% of patients in 2020 presented to the hospital without ambulance transport, compared to 81.1% of patients in the 2021 non-COVID-19 pandemic period. Patients with NIHSS scores of 6–14 were more likely to call an ambulance for transport to the hospital than to transport themselves to the emergency department.

**Conclusions:**

During the 2020 COVID-19 pandemic, the DNT was prolonged as a result of strengthened fever surveillance. In 2021, the number of patients with acute ischemic stroke who underwent ITT increased compared to the previous year. Notably, the growth in the number of patients with acute ischemic stroke who underwent ITT benefited from both the “stroke emergency map” of China and the “green channel,” a novel treatment approach that focuses on the rational design of the rescue process.

Trial registration.

Our study was a retrospective descriptive study, not a clinical trial, thus we did not have to register for clinical trials.

## Background

Stroke is characterized by high rates of morbidity, disability, mortality, and recurrence as well as a high economic burden [1]. Treatment outcomes are time-dependent because brain cells die if blood flow to the brain is completely blocked for 5 min. With every minute of delay, 1.9 million neurons die [2], potentially causing irreversible damage. This is particularly true in patients with ischemic stroke. For every 30-min delay in reperfusion, the probability of a “good” 90-day prognosis decreases by approximately 12% [3]. Intravenous thrombolytic therapy (ITT) within 6 h of onset is recommended for acute ischemic stroke (AIS) and is globally recognized as one of the only effective rescue methods for reducing disability due to stroke [4].

In China, the city of Dalian released the first batch of “stroke emergency maps” in July 2019, covering 13 hospitals, with Dalian Municipal Central Hospital acting as a map management hospital. The 13 hospitals were located in central Dalian and included surrounding secondary and tertiary hospitals. These hospitals provide emergency stroke treatment and are equipped with intravenous thrombolysis technology. A “stroke emergency map” is a patient resource that offers 4 shortcut functions: 1. early stroke recognition, 2. dialing 120 for emergency medical services with a one-click call function, 3. creating a stroke emergency card with personal information, and 4. navigating to the nearest hospital. Consequently, this resource is of great importance for patients seeking medical care following a stroke. Stroke patients can navigate to the nearest suitably equipped hospital for treatment using the map obtained from the official “Stroke Map” WeChat account, or they can dial 120 for emergency medical services with a one-click call function. After the introduction of stroke emergency maps, the “green channel” was opened. This new clinical treatment strategy focuses on the rational design of the rescue processes. All the diagnostic and treatment data of AIS patients within the thrombolytic therapy time window at the 13 participating hospitals were uploaded to the map application platform of the National Cerebral Defense Commission. This system was fully functional during the coronavirus disease 2019 (COVID-19) pandemic of 2020. We collected intravenous thrombolysis data from 13 hospitals in Dalian from February to August 2020 and compared them with data from the corresponding period in 2021 to explore the impact of the COVID-19 pandemic on emergency stroke treatment and the “green channel.”

## Methods

### Study patients

From the 13 hospitals in Dalian, China, that used the stroke map, we collected data pertaining to the diagnosis and treatment of all patients with AIS who underwent intravenous thrombolysis between February 1, 2020, and August 31, 2020 (during the COVID-19 pandemic), and during the corresponding time period in 2021. The 13 hospitals had special map management quality control teams and personnel responsible for monthly data quality control and reporting. The data included the number of patients who underwent intravenous thrombolysis, the onset-to-door time (ODT), the door-to-needle time (DNT), the onset-to-needle time (ONT), the means of transportation to the hospital, and the National Institutes of Health Stroke Scale (NIHSS) scores before and after ITT. Data were reported to the National Cerebral Defense Commission through a stroke emergency map data platform network(Stroke Emergency Map Management System of China). Data from 2020 were compared with those collected during the corresponding period (between February 1 and August 31) in 2021 (Table 1).

**Table 1:** Comparison of AIS patients receiving intravenous thrombolysis in 2020 and 2021

Characteristics	2020 (*n* = 735)	2021 (*n* = 1719)	U/χ^2^	*P* value
Sex [n (%)]				
Male	507 (69.0)	1122 (65.3)	3.174	0.075
Female	228 (31.0)	597 (34.7)		
Age (years, median (P25, P75)	68 (61, 74)	67 (59, 75)	611,432	0.207
Mode of transportation to the hospital [n (%)]				
No ambulance support	617 (83.9)	1394 (81.1)	8.695	0.034
Nosocomial attacks	2 (0.3)	20 (1.2)		
"120″ ambulance services	107 (14.6)	293 (17)		
Transferred from other hospitals	9 (1.2)	12 (0.7)		
Before thrombolysis NIHSS (score, median (P25, P50))	5 (3, 11)	5 (2, 10)	598,775	0.040
ODT (min, median (P25, P75))	100 (60, 155)	112 (61, 160)	595,541	0.024
ONT (min, median (P25, P75))	165 (127, 225)	160 (116, 210)	581,733	0.002
DNT (min, median (P25, P75))	59 (44, 80)	45 (32, 59)	419,387	< 0.001
Name of thrombolytic drug [n(%)]				
Urokinase	121 (16.5)	266 (15.5)	0.379	0.538
Alteplase	614 (83.5)	1453 (84.5)		

*AIS* Acute ischemic stroke, *NIHSS* National Institutes of Health Stroke Scale, *ODT* onset-to-door time, *DNT* Door-to-needle time, *ONT* Onset-to-needle time

### Ethics approval

This study was approved by the ethics committee of the Dalian Municipal Central Hospital (approval number: 2021–054-01). The requirement for informed consent was waived because of the retrospective, descriptive design of the study.

### Patient grouping

We divided the patients into two groups according to their mode of transport to the hospital: by their own means (*n* = 617 in 2020, *n* = 1394 in 2021) or by emergency (“120”) ambulance transport (*n* = 107 in 2020, *n* = 293 in 2021). Each group was further divided according to the NIHSS scores obtained immediately before thrombolysis, as follows: self-transported patients who scored ≤ 5 points (*n* = 351 in 2020, *n* = 818 in 2021), 6–14 points (*n* = 182 in 2020, *n* = 438 in 2021), or ≥ 15 points (*n* = 84 in 2020, *n* = 138 in 2021) and ambulance-transported patients who scored ≤ 5 points (*n* = 29 in 2020, *n* = 90 in 2021), 6–14 points (*n* = 35 in 2020, *n* = 125), or ≥ 15 points (*n* = 43 in 2020, *n* = 78 in 2021). We then evaluated the differences in NIHSS scores before and immediately after thrombolysis (Table 2).

**Table 2:** NIHSS score comparison before and after ITT among AIS patients by hospital transportation mode during corresponding periods in 2020 and 2021

Before thrombolysis NIHSS score	Mode of transportation to the hospital	2020 (*n* = 724)				2021 (*n* = 1687)			
		N (%)	Change in NIHSS score after thrombolysis		*P* value	N (%)	Change in NIHSS score after thrombolysis		*P* value
			Decrease or no change	Increase			Decrease or no change	Increase	
≤ 5 points	No ambulance support	351 (56.9)	341 (97.2)	10 (2.8)	< 0.001	818 (58.7)	792 (96.8)	26 (3.2)	< 0.001
	"120″ ambulance services	29 (27.1)	29 (100)	0 (0)	0.016	90 (30.7)	87 (96.7)	3 (3.3)	< 0.001
6–14 points	No ambulance support	182 (29.5)	177 (97.3)	5 (2.7)	< 0.001	438 (31.4)	429 (97.9)	9 (2.1)	< 0.001
	"120″ ambulance service	35 (32.7)	30 (85.7)	5 (14.3)	0.5455	125 (42.7)	121 (96.8)	4 (3.2)	< 0.001
≥ 15 points	No ambulance support	84 (13.6)	82 (97.6)	2 (2.4)	< 0.001	138 (9.9)	135 (97.8)	3 (2.2)	< 0.001
	"120″ ambulance services	43 (40.2)	40 (93)	3 (7)	0.012	78 (26.6)	74 (94.9)	4 (5.1)	< 0.001

*NIHSS* National Institutes of Health Stroke Scale, *ITT* Intravenous thrombolytic therapy, *AIS* Acute ischemic stroke

### Statistical analyses

SPSS software (version 23.0; IBM Corp., Armonk, NY, USA) and the Microsoft Office Excel Data Analysis ToolPak (Microsoft Corp., Redmond, Washington, USA) were used for the statistical analyses. The Kolmogorov‒Smirnov test was used to test the normality of measurement data, and the results showed that none of the items conformed to a normal distribution. Data are expressed as the median (P25, P75), and the Mann‒Whitney U test was used for comparisons between groups. Comparisons of NIHSS scores before and after thrombolysis were performed using the Wilcoxon signed-rank test. Count data are expressed as N (%) and were analyzed using the χ^2^ test or Fisher’s exact test. Statistical significance was set at *P* < 0.05.

## Results

### Comparison of AIS patients receiving intravenous thrombolysis

In 2020, the total number of patients who went to the hospital within 6 h of AIS onset was 1626, of whom 735 (45.2%) were treated with ITT and 891 (54.8%) were not. However, in 2021, the total number of such patients was 2451, of whom 1719 (70.3%) were treated with ITT and 732 (29.9%) were not. The number of patients with AIS who received intravenous thrombolysis significantly increased by 133.9% from 2020 to 2021. The majority of the patients were men. Overall, the patients ranged in age from 24 to 98 years (24–95 years in 2020, 27–98 years in 2021). Patients who presented to the hospital without ambulance transport accounted for > 80% of patients in both groups. Most intravenous thrombolysis therapies were performed using alteplase (83.5% in 2020 and 84.5% in 2021). Significant differences in ONT, ODT and median DNT were observed (*P* < 0.05) during the corresponding periods of the two examined years. The median NIHSS score was five points in both groups (Table 1). Compared with the data from 2020, the number of patients with AIS who underwent ITT increased in 2021, but the DNT decreased.

### Comparison of the change in NIHSS scores after intravenous thrombolysis among patients with AIS by mode of transportation to the hospital

Patients who called “120” for ambulance transport demonstrated significantly higher NIHSS scores than those who arrived at the hospital without ambulance transport. Most patients with NIHSS scores ≤ 5 points came to the hospital without ambulance transport, whereas most patients with NIHSS scores ≥ 6 points called “120” for an ambulance to the hospital. Comparisons were made between patients whose NIHSS scores in the two groups in 2020 and 2021 before and immediately after thrombolysis were ≤ 5 points and ≥ 15 points, respectively, and a significant difference was observed; the same was true of patients with a score of 6–14 points in 2021 (Table 2). Therefore, the higher the NIHSS score, the more likely a patient was to call “120” for ambulance transport to the hospital. Most patients benefit from intravenous thrombolysis within the first 6 h of AIS.

## Discussion

In 2020, the COVID-19 pandemic affected health care procedures globally, including the diagnosis and treatment of acute strokes. Two outbreaks occurred in Dalian, one on July 22 and the other on December 15, 2020, eliciting an enormous governmental response to control the spread of the disease. AIS is a common cerebrovascular disease whose treatment is aimed at promptly re-establishing cerebral perfusion and avoiding ischemic necrosis within the ischemic penumbra [5]. Therefore, the outcomes are highly time dependent. During the 2020 COVID-19 pandemic, epidemiological investigation and screening, among other factors, delayed the diagnosis and treatment of several patients with AIS.

The number of patients with AIS who underwent intravenous thrombolysis was significantly higher in 2021 (133.9%) than in the same period in 2020. However, the treatment outcomes of patients with AIS who underwent intravenous thrombolysis were unaffected by the COVID-19 pandemic. This is likely related to the strengthening of stroke health management, popular knowledge of science, and education provided by the National Brain Prevention Commission as well as by government departments and medical practitioners at all levels. Through initial adherence to publicity and educational efforts, stroke awareness among patients and their family members increased, as did the treatment rates.

When we compared patients from corresponding periods in two different years, we observed no significant differences in age, sex, or intravenous thrombolytic drugs. However, in 2020, DNT was prolonged, exceeding the average DNT of 45 min in China and the United States [6]. In addition to the signing of informed consent and other procedures [7], this increase was related to the heightened surveillance of fever; epidemiological history investigations; and screening for symptoms associated with respiratory disease, such as cough, sputum production, chest tightness, diarrhea, and fatigue, during the COVID-19 pandemic. This in-hospital delay could potentially have been shortened by 20 min if the requirement for informed consent had been waived [8]. Nevertheless, in the context of standardized disease prevention and control, we must consider how else we can shorten the in-hospital processing time of patients with AIS [9].

The stroke awareness rate was not as high in 2020 as that in 2021. Fewer rural AIS patients and a shorter ODT were observed in 2020 than in 2021. However, due to efficient public information, stroke is a well-known topic to health care consumers. The rate of early stroke recognition increased from 2020 to 2021, as did the rate of using “stroke maps” to visit nearby hospitals. At the same time, when the number of rural patients increased, the scope of medical treatment expanded. However, rural patients needed time to travel from the countryside to the nearest suitably equipped hospital; therefore, the ODT increased in 2021, which urges us to further improve the role of stroke maps and simultaneously improve the stroke emergency capacity of rural hospitals.

Regardless of the pandemic, AIS patients with an NIHSS score ≤ 5 were more likely to use their own vehicles to reach the hospital than to be transported by ambulance. This is likely related to the recent increase in car ownership in China, as reported by the Ministry of Public Security. Moreover, the stroke emergency map can be navigated such that patients can easily proceed to the nearest hospital with the appropriate treatment capacity. Moreover, self-transport was used not only to avoid an uncertain waiting period for an ambulance but also to avoid concerns regarding the sanitization of ambulances during the pandemic [9]. In contrast, patients with NIHSS scores ≥ 6 points were more likely to opt for emergency medical transport via the “120” rescue system.

Statistically significant differences were observed between the values: the NIHSS scores of the two groups in 2020 and 2021 before and immediately after thrombolysis were ≤ 5 and ≥ 15 points, and the NIHSS scores were 6–14 points in 2021. This indicates that more patients with AIS benefited from therapy. Such patients need to be followed-up for 1, 3, and 12 months after stroke onset to verify their prognoses.

This study has some limitations. First, the follow-up period in the present study was short. Further studies with additional timepoints such as 30, 60 and 90 days are needed to confirm these results. Second, the design of this study was retrospective and descriptive; therefore, our findings should be verified through prospective clinical studies. Third, all the patients who underwent intravenous thrombolysis required additional follow-up.

In summary, timely management is crucial for optimizing treatment outcomes in patients with AIS [10]. With standard prevention and control measures in place for the COVID-19 pandemic, stroke emergency maps should be actively promoted to allow patients to choose the nearest hospital that can provide emergency treatment, whether they reach the hospital without ambulance transport or by calling “120” for assistance. Hospitals should establish efficient, high-speed green channels to help shorten the DNT, provide additional time for intravenous thrombolysis, and reduce stroke death and disability rates.

## Data Availability

The datasets used and/or analyzed during the current study are available from the corresponding author on reasonable request.

## Abbreviations

COVID-19: Coronavirus disease 2019
DNT: Door-to-needle time
ODT: Onset-to-door time
ONT: Onset-to-needle time
NIHSS: National Institutes of Health Stroke Scale
AIS: Acute ischemic stroke
